# Change in taste sensation after orthognathic surgery

**DOI:** 10.1007/s00784-024-05626-1

**Published:** 2024-04-01

**Authors:** Yagmur Malkoc, Serap Gulsever, Sina Uckan

**Affiliations:** 1 Graduate School of Health Sciences, School of Dentistry, İstanbul Medipol University, Atatürk Bulvarı No:27, 34083 Unkapanı, Fatih, İstanbul Türkiye; 2 Department of Oral and Maxillofacial Surgery, School of Dentistry, İstanbul Medipol University, TEM Avrupa Otoyolu Göztepe Çıkışı, 34214 Bağcılar, İstanbul Türkiye

**Keywords:** Orthognathic surgery, Le Fort I Osteotomy, Sagittal Split Ramus Osteotomy, Gustation, Taste sense

## Abstract

**Objective:**

The objective of this study was to evaluate the effect of orthognathic surgery on taste sensation.

**Materials and methods:**

Thirty-five patients scheduled to undergo Le Fort I osteotomy (LFIO), sagittal split ramus osteotomy (SSRO), and bimaxillary surgery (BMS) were evaluated by administering localized and whole-mouth taste tests preoperatively and postoperatively at months 1, 3, and 6. The patients were asked to identify the quality of four basic tastes applied to six locations on the palate and tongue and to rate the taste intensities they perceived. Taste recognition thresholds and taste intesity scores were evaluted according to operation groups and follow-ups.

**Results:**

There were significant decreases in the *quinine HCl* recognition thresholds at the postoperative follow-ups compared to the preoperative in LFIO patients (*p* = 0.043). There were significant decreases in *sucrose* taste intensity scores in the right posterolateral part of the tongue at months 3 and 6 compared to preoperative in SSRO patients (*p* = 0.046), and significant increases in *quinine HCL* taste intensity scores in the right and left anterior parts of the tongue at month 6 compared to preoperative in LFIO patients (*p* < 0.05).

**Conclusion:**

Taste perception is affected due to potential damage to the chemosensory nerves during orthognathic surgical procedures. Generally, non-significant alterations have been observed in taste perception after orthognathic surgery, except for significant alterations in bitter and sweet taste perceptions.

**Clinical relevance:**

Maxillofacial surgeons should be aware of taste perception change after orthognathic surgery procedures and patients should be informed accordingly.

**The trial registration number (TRN):**

NCT06103422/Date of registration: 10.17.2023 (retrospectively registered).

## Introduction

Different taste modalities are perceived by the taste buds located on the oral mucosa, palate, tongue, and pharynx. The stimulation of taste receptors results in the transmission of signals to the central nervous system via afferent neurons of the cranial nerves. Taste sensation from the anterior two-thirds of the tongue is carried by the parasympathetic fibers of the chorda tympani, a branch of the facial nerve that joins the lingual nerve while taste sensation from the posterior one-third is carried by the glossopharyngeal nerve. The chorda tympani nerve runs in the lingual soft tissue in the mandibular posterior region and extends toward the floor of the mouth. Taste sensation from the soft palate is carried by the major superficial petrosal nerve, a branch of the facial nerve, which travels in two branches of the palatine nerve [1].

Craniofacial nerve conduction may be affected after orthognathic surgical procedures performed to correct craniofacial deformities. Somatosensory and gustatory deficits associated with Le Fort I osteotomy (LFIO) and sagittal split ramus osteotomy (SSRO) have been documented in the literature [2–5]. SSRO procedures may cause direct trauma to the lingual and inferior alveolar nerves or indirect damage due to edema, pressure of fixation devices, compression, and stretching [6–8]. Moreover, because the chorda tympani innervate parasympathetic fibers to the submandibular and sublingual glands, damage to fibers may impair gustation by diminishing salivary flow [9]. While some branches of the trigeminal nerve are always divided during LFIO, some branches (palatine nerve, infraorbital nerve) are at risk of damage due to stretching, retraction, pressure, and plate-screw fixation [5].

Problems that develop as a result of the change in the sense of taste, such as dislike of some foods, malnutrition, weight loss, decreased quality of life, and depression, may prolong and complicate the postoperative care of orthognathic surgery patients [10]. Several studies have evaluated gustation changes after the surgical extraction of impacted wisdom teeth [11, 12], nasal surgical procedures [13], and local anesthesia applications performed in the innervation area of these nerves [14, 15]. However, to our knowledge, there are only two previous studies in the literature that evaluated changes in taste sensation after orthognathic surgical procedures, conducted with a small sample size [3, 4]. Information that will be obtained through clinical studies with greater sample sizes regarding the risk of possible complete or partial taste perception loss after orthognathic surgery will provide the opportunity to inform patients about potential postoperative somatosensorial changes in the preoperative period.

This study aimed to evaluate the severity and time course of changes in taste sensation via the localized and whole-mouth taste tests in patients who underwent orthognathic surgery.

## Materials and methods

### Subjects

This prospective study was approved by the İstanbul Medipol University Institutional Review Board and Ethics Committee (ethical approval no: 10840098-604.01.01-E.16461) and was conducted in accordance with World Medical Association Declaration of Helsinki of 1975 as revised in 2000. Written informed consent was obtained from each patient prior to the commencement of the study.

Power analysis was implemented before the data collection process, using G*Power software. The sample size was calculated as 28 using the simple random sampling method (p=0.5; q=0.5; α=.05).

The subjects were the patients with Class II or III dentofacial deformities who applied to the Department of Oral and Maxillofacial Surgery between July 2020 and December 2021. Patients with an ASA status III and above; those with zinc, iron, and/or other vitamin deficiencies known to affect the sense of taste; smokers; those with complex craniofacial syndromes; and those with a history of chemotherapy and radiotherapy to the head and neck region, orthognathic surgery, maxillofacial trauma, or damage to the nerves related to the taste sensation were excluded.

The same surgical team performed all operations. LFIO procedures were performed according to Bell modification and SSRO procedures were performed according to the Hunsuck-Epker modification under general anesthesia. In the LFIO patients, following the mucoperiosteal incision and dissection osteotomies were performed with a reciprocal saw from the pterygomaxillary junction to the apertura piriformis. In the SSRO patients, the osteotomies were performed from the lingula of the mandible to the mandibular base at the second molar level. 2.0 miniplate system was used for fixation (KLS Martin®, Germany). In the postoperative period, antibiotics, analgesics, antipyretics, and antiemetics were administered parenterally during the hospitalization.

The primary outcome measure was the taste recognition threshold, and the secondary outcome measure was the taste intensity score. Alterations in taste recognition thresholds and taste intensity scores were evaluated between the operation groups and between the follow-ups within the operation groups.

### Taste tests

Gustatory functions were evaluated by administering the whole mouth taste test (*WMTT*) and localized taste test (*LTT)* preoperatively (T0) and postoperatively at months 1 (T1), 3 (T2), and 6 (T3). The patients were informed to stop their food intake for two hours before the test. The mouth was rinsed with distilled water before administering each series of different flavor solutions, and the waiting time between applications was one minute. The taste solutions, acquired from Merck (Darmstadt, Germany), were applied by the same clinician in the order of sodium chloride (NaCl), *sucrose*, citric acid, and quinine hydrochloride (HCl). The solutions contained *sucrose* 300 mg/ml to represent the sweet taste, NaCl 80 mg/ml for the salty taste, citric acid 60 mg/ml for the sour taste, and *quinine HCl* 1 mmol/L for the bitter taste [11, 13]. The solutions were prepared at regular intervals by the Department of Pharmacy of Medipol University and applied at room temperature (22–24°C).

#### Whole mouth taste test

In the WMTT, five concentration levels (in ½ log steps) of the solutions were prepared in 1 ml solution samples. One ml of the specific solution with the lowest concentration was drawn into a syringe and sprayed into the mouth of the patient circularly. The solutions were administered in increasing concentrations, starting from the lowest concentration (C1) to the highest concentration (C5) until the patient perceived any taste. After each administration, the patients spat the solution out and reported whether they had perceived any taste and, if so, which taste. The lowest concentration at which the patient correctly perceived the administered taste was defined as the taste recognition threshold. If the patient could not perceive the correct taste even with the application of the highest concentration of a solution, the recognition threshold was recorded as C6 for the applied taste. The taste series were applied in the same way for four basic taste modalities [11, 13].

#### Localized taste test

In the LTT, the highest concentration solution of one of the four tastes used in the WMTT was administered. In each application, 0.25 ml of solution was absorbed on a sterile cotton swab and applied to six test areas on the palate and tongue. The patients were instructed to focus on the perceived taste without closing their mouths and asked to identify the taste and rate the intensity of the taste using a scale ranging from 0 (no taste) to 9 (strongest taste) after each administration [11, 13]. The first tested locations were the right and left anterior (chorda tympani nerve receptive area) and posterolateral surfaces of the tongue (glossopharyngeal nerve receptive area). The locations tested next were the right and left sides of the soft palate lateral to the midline (greater superficial petrosal nerve receptive area).

### Statistical analysis

The Statistical Package for the Social Sciences version 26 was used for the statistical analysis. Descriptive statistical methods (i.e., mean, standard deviation, median, frequency, percentage, minimum, and maximum) were used to evaluate the data. The conformity of the quantitative data to normal distribution was assessed using the Shapiro–Wilk test and graphical analysis. The Kruskal–Wallis and Dunn–Bonferroni tests were used to compare the non-normally distributed quantitative variables between more than two groups. The Friedman test was used for intra-group comparisons of the non-normally distributed quantitative variables, and the Wilcoxon signed-rank test with Bonferroni correction was used to evaluate the pairwise comparisons. The Fisher–Freeman–Halton test was used to compare the qualitative data. Statistical significance was accepted as *p* < 0.05.

## Results

### Demographic and descriptive data

Of the 52 patients included in this clinical study, 16 were excluded for reasons such as drug use and loss of contact during the six-month follow-up period, and one due to the need for revision surgery. Eight (22.9%) of the patients underwent LFIO, five (14.3%) SSRO, and 22 (62.9%) BMS. Among the patients, 13 (37.1%) were male and 22 (62.9%) female. The ages of the patients ranged from 17 to 42 years, and there was no statistically significant difference between the mean ages of the groups. The ratio of the Skeletal Class II relationship was significantly higher in the patients who underwent SSRO than in the other surgical groups (*p* = 0.007) (Table 1).

**Table 1:** Demographic and descriptive data

		LFIO	SSRO	BMS	*p*
		*n* (%)	*n* (%)	*n* (%)	
Gender	Male	4 (50.0)	1 (20.0)	8 (36.4)	^***a***^***0.609***
	Female	4 (50.0)	4 (80.0)	14 (63.6)	
Skeletal Class	Class II	0 (0.0)	4 (8.0)	6 (27.3)	^***a***^0.007*
	Class III	8 (100.0)	1 (20.0)	16 (72.7)	
Age (years)	Mean ± SD	26.38 ± 7.76	24.60 ± 2.88	26.73 ± 7.45	^***b***^***0.882***
	Median (Min.-Max.)	24.5 (19–42)	25 (20–28)	27 (17–40)	

^*a*^*Fisher Freeman Halton Test*

^*b*^*Kruskal Wallis Test*

*p < *0.05*

### Whole mouth taste test

**Fig. 1:**
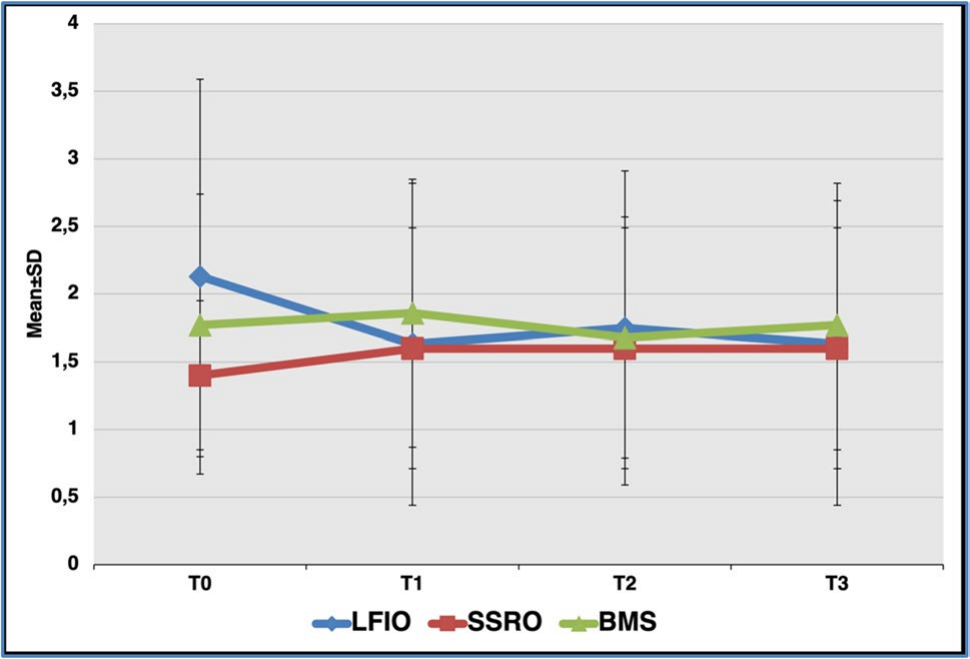
The distribution of the mean taste recognition thresholds for *quinine HCl* determined by whole mouth taste test

There were no statistically significant differences between all the operation groups in terms of mean *sucrose*, NaCl, citric acid, and *quinine HCl* recognition thresholds at all follow-up times. In addition, no statistically significant differences were observed between the follow-up periods with respect to the mean taste recognition thresholds for any of the taste compounds in the BMS and SSRO groups. In the LFIO group, no significant differences were noted for the sweet, salty, and sour recognition thresholds between the follow-up periods, whereas the bitter recognition thresholds were significantly lower in the postoperative follow-ups compared to the T0 period (*p* = 0.043) (Fig. 1).

### Localized taste test

**Fig. 2:**
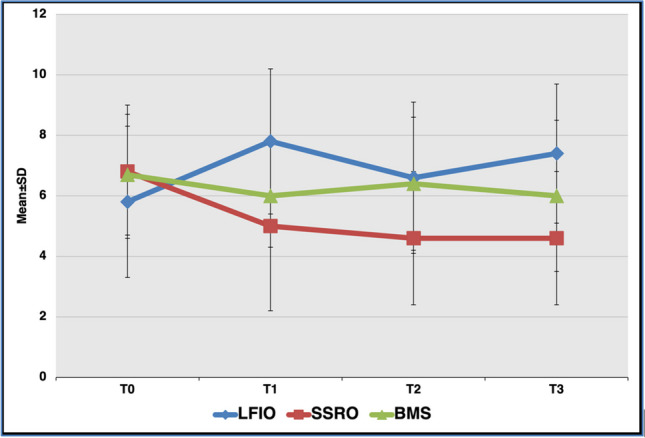
The distribution of the mean taste intensity scores of the right posterolateral region of the tongue for *sucrose* determined by localized taste test

For the *sucrose* taste intensity scores, the differences between the groups for each test location of the tongue at T1 were statistically significant (right anterior tongue, *p* = 0.042; left anterior tongue, *p* = 0.044; right posterolateral tongue, *p* = 0.047; left posterolateral tongue, *p* = 0.046), while the differences were not significant for the right and left soft palate at any time. In the SSRO group, the *sucrose* intensity scores in the right posterolateral part of the tongue at T2 and T3 were significantly lower than at T0 (*p* = 0.046 and *p* = 0.046, respectively), whereas the differences between all the other visits within the groups were not significant for any of the other test locations (Fig. 2).

For NaCl taste intensity scores, the differences between the operation groups in the anterior right and left of the tongue at T0 were statistically significant (*p* = 0.012)*,* while the differences between the groups in all other regions and at all other follow-ups were not significant. The differences between all follow-ups within all operation groups were not significant for all test locations.

**Fig. 3:**
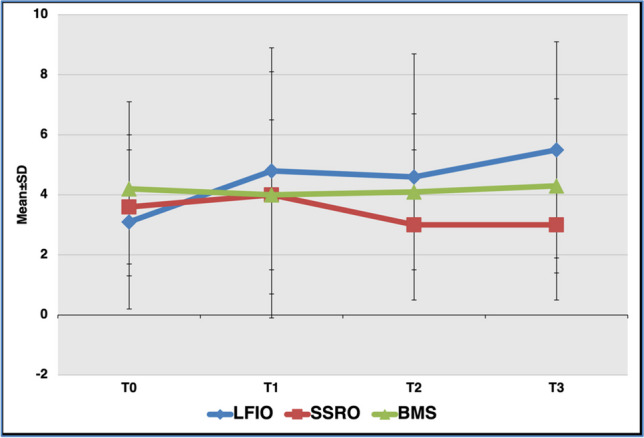
The distribution of the mean taste intensity scores of the right anterior region of the tongue for *quinine HCl* determined by localized taste test

**Fig. 4:**
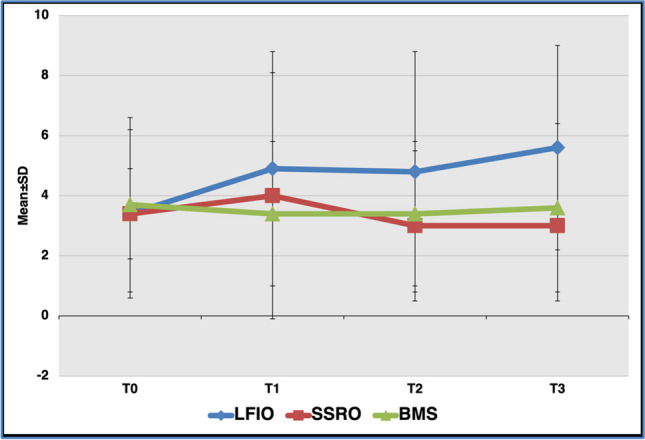
The distribution of the mean taste intensity scores of the left anterior region of the tongue for *quinine HCl* determined by localized taste test

For citric acid taste intensity scores, there were no significant differences between the operation groups. The *quinine HCl* taste intensity scores in the right and left anterior of the tongue at T3 were significantly higher than at T0 in the LFIO group (*p* = 0.039 and *p* = 0.014, respectively) (Figs. 3 and  4).

## Discussion

Craniofacial nerves may be injured by compression, laceration, traction, or penetrating mechanisms due to mechanical trauma caused by the instruments used in orthognathic surgery procedures [16, 17]. This nerve damage is influenced by factors such as the duration and type of trauma, the magnitude of the applied force, the diameter of the nerve fiber, the location of the nerve fascicle, and the nerve regenerative capacity [18]. In addition, the heat caused by the tour engines and electric cautery systems may cause thermal damage to the nerve tissue and surrounding soft tissues [19].

Various examination methods have been used to evaluate taste function. Taste strips, filter paper discs, taste tablets, and taste solution tests are frequently used in clinical practice to assess sensitivity to the four main taste modalities [20, 21]. Taste strips and filter paper discs are semi-quantitative, quick, and simple taste function assessment tools and have a long shelf life. The limitations to the use of both methods are non-quantitative data and procurement difficulties. Another method is electrogustometry (EGM), which is used to assess detection thresholds. Although EGM is a quantitative, accurate, fast, and effortless test tool, it creates a metallic/sour taste sensation and is not dependent on the identification of taste qualities [22, 23]. In this study, taste solutions were used due to the ease of preparation, portability, long shelf life, quick applicability, and reproducibility of the measurement.

In the WMTT, five solutions of increasing concentrations were applied for each of the sweet, salty, bitter, and sour tastes. While the application of different concentrations increases the sensitivity of the technique in determining a patient’s recognition threshold, repeated applications may cause an increase in the recognition threshold as a result of desensitization of the taste buds [24, 25]. Since it is not possible to identify specifically damaged nerves by performing WMTT, LTT was also performed in this study.

Somatosensory changes after orthognathic surgery have been evaluated in numerous studies in the literature; however, only two studies have assessed taste perception changes [3, 4]. Sanger [4] evaluated the olfactory and gustatory function in three patients who underwent BMS and two patients who underwent isolated SSRO. LTTs and WMTTs were performed by applying 25 solutions at varying concentrations of five different substances for the bitter, sour, sweet, and salty tastes two weeks before the surgery and postoperatively at months 2 and 6. The WMTT scores for the salty, sweet, and sour tastes significantly decreased two months after surgery; however, six months after surgery these scores remained unchanged in two patients but exceeded their preoperative values in the other two patients. Similarly, in our study, bitter taste intensity scores in the right and left anterior tongue regions in LFIO patients exceeded the preoperative value at the six-month postoperative follow-up.

Taste buds are specific to a particular taste but are distributed throughout the mouth cavity so even if regional differences are apparent in taste acuity, all four basic taste qualities can be detected throughout the mouth [25]. We assumed that after LFIO, the hypergeusia in the anterior part of the tongue that occurred in the perception of the bitter taste may have been due to the hyperfunction of the taste buds in the anterior region of the tongue, which are sensitive to the bitter taste. This hyperfunction may have resulted from an attempt to compensate for the sensation loss in the soft palate and pharynx caused by potential damage to the peripheral nerves that conduct afferent chemosensory information from these regions. Such damage may occur during the manipulation of instruments such as an endotracheal tube, laryngoscope, or Magill forceps, or during surgical maneuvers such as cauterization, corticotomy, retraction, or due to postoperative hematoma and edema.

In a study by Gent et al, the effects of orthognathic surgery on the taste function of the palate and tongue were examined via an LTT, which was performed at the preoperative visit and one to two months and six to nine months postoperatively [3]. Of the nine patients, five underwent SSRO only, three BMS, and one LFIO only. The authors observed a significant decrease in the postoperative perception of all flavors in general, although regional changes were observed depending on the type of surgery [3]. They reported that at the postoperative visits, the perceived taste intensity on the palate decreased by 34% compared to preoperatively for NaCl, *sucrose*, and citric acid after LFIO. Furthermore, the average correct identification of tastes by the LFIO patients decreased from 91% to 38%, and the taste intensity scores for quinine on the tongue decreased to 72% of their preoperative values following SSRO. In our study, although in general non-significant alterations have been observed in taste perception with some exceptions, the results regarding the change in taste perception on the palate after LFIO were comparable to those observed in the study by Gent et al. In the patients who underwent LFIO and BMS, the taste intensity ratings and correct taste quality identifications of all the flavors on both the right and left soft palate were reduced at the postoperative visits. On the other hand, we observed a significant decrease in the taste intensity ratings of *sucrose* perceived on the right posterolateral part of the tongue at the months 3 and 6 postoperative visits in the patients who underwent SSRO. These differences may be attributed to the small patient population in Gent et al.'s study, the use of bupivacaine, which is known to have a neurotoxic effect, as a local anesthetic agent, and mandibular fixation with three bicortical positional screws, which are known to have a high risk of lingual nerve damage [7] and infection [8]. In our study, Lidocaine 20 mg/mL with epinephrine 0.0125 mg/mL was used as a local anesthetic agent, and mandibular fixation was performed using the miniplate and monocortical screws on either side.

Depending on the degree of injury, neurological recovery after peripheral nerve damage may occur via mechanisms such as remyelination, collateral axon sprouting, and regeneration from the proximal site. In neuropraxia, remyelination takes place within 2–12 weeks, in limited or moderate axonal loss, collateral sprouting and axonal regeneration within 2–6 months, and in severe axonal loss, axonal regeneration occurs within 2–18 months [26]. Unlike Sanger and Gent et al., we subjected our patients to three follow-ups to observe the possible changes in nerve regeneration within six months postoperatively at shorter time intervals. In addition, all the tests were carried out prior to surgery to identify any existing problems or individual variations in taste perception.

The loss of receptors observed with aging throughout the lifespan results in the loss of sensitivity in the senses of taste and smell; however, the sense of smell is more affected [27]. Since the patients included in our study were young adults aged 17–42 years, age-related differences in taste perception between the individuals were not expected.

The limitations of this study were the inability to evaluate the complete recovery in taste perception alterations because severe nerve injuries require longer recovery times and the lack of evaluation of the effects of the direction and amount of maxillary/mandibular movement on taste perception. Further studies with larger samples and longer follow-up periods that consider the effects of the amount and direction of bone movement on taste perception are required.

## Conclusion

Following orthognathic surgery, taste perception is affected possibly by damage to peripheral nerves that transmit chemosensory information. With some exceptions, non-significant alterations were observed in taste perception following orthognathic surgical procedures. Minor losses in taste perception were regained after six months following surgery. According to the findings from WMTT and LTT, an increase in bitter taste perception was observed after Le Fort I osteotomy.

## Data Availability

No datasets were generated or analysed during the current study.

## Declarations

This study is Yağmur Malkoç's doctorate thesis.
